# The Stimulatory Adenosine Receptor ADORA2B Regulates Serotonin (5-HT) Synthesis and Release in Oxygen-Depleted EC Cells in Inflammatory Bowel Disease

**DOI:** 10.1371/journal.pone.0062607

**Published:** 2013-04-26

**Authors:** Rikard Damen, Martin Haugen, Bernhard Svejda, Daniele Alaimo, Oystein Brenna, Roswitha Pfragner, Bjorn I. Gustafsson, Mark Kidd

**Affiliations:** 1 Gastrointestinal Pathobiology Research Group, Yale University School of Medicine, New Haven, Connecticut, United States of America; 2 Department of Cancer Research and Molecular Medicine, Norwegian University of Science and Technology, Trondheim, Norway; 3 Institute of Pathophysiology and Immunology, Centre for Molecular Medicine, Graz, Austria; Duke University Medical Center, United States of America

## Abstract

**Objective:**

We recently demonstrated that hypoxia, a key feature of IBD, increases enterochromaffin (EC) cell 5-HT secretion, which is also physiologically regulated by the ADORA2B mechanoreceptor. Since hypoxia is associated with increased extracellular adenosine, we wanted to examine whether this nucleotide amplifies HIF-1α-mediated 5-HT secretion.

**Design:**

The effects of hypoxia were studied on IBD mucosa, isolated IBD-EC cells, isolated normal EC cells and the EC cell tumor derived cell line KRJ-1. Hypoxia (0.5% O_2_) was compared to NECA (adenosine agonist), MRS1754 (ADORA2B receptor antagonist) and SCH442146 (ADORA2A antagonist) on HIF signaling and 5-HT secretion. Antisense approaches were used to mechanistically evaluate EC cells *in vitro*. PCR and western blot were used to analyze transcript and protein levels of HIF-1α signaling and neuroendocrine cell function. An animal model of colitis was evaluated to confirm hypoxia:adenosine signaling *in vivo*.

**Results:**

HIF-1α is upregulated in IBD mucosa and IBD-EC cells, the majority (∼90%) of which express an activated phenotype *in situ*. Hypoxia stimulated 5-HT release maximally at 30 mins, an effect amplified by NECA and selectively inhibited by MRS1754, through phosphorylation of TPH-1 and activation of VMAT-1. Transient transfection with *Renilla* luciferase under hypoxia transcriptional response element (HRE) control identified that ADORA2B activated HIF-1α signaling under hypoxic conditions. Additional signaling pathways associated with hypoxia:adenosine included MAP kinase and CREB. Antisense approaches mechanistically confirmed that ADORA2B signaling was linked to these pathways and 5-HT release under hypoxic conditions. Hypoxia:adenosine activation which could be reversed by 5′-ASA treatment was confirmed in a TNBS-model.

**Conclusion:**

Hypoxia induced 5-HT synthesis and secretion is amplified by ADORA2B signaling via MAPK/CREB and TPH-1 activation. Targeting ADORA2s may decrease EC cell 5-HT production and secretion in IBD.

## Introduction

Inflammatory Bowel Disease (IBD) is highly prevalent in Europe and North America and a recent systematic review demonstrated an increasing incidence (for UC: 6.3–24.3/100,000; for CD: 5–20.2) [1]. This coupled with the long duration of the illness make IBD one of the most common gastroenterological diseases with a prevalence per 100,000 of 505 and 249 for UC and 322 and 319 for CD in Europe and the US, respectively [1]. The etiology and pathogenesis of IBD, however, remains largely unknown. While defects in local immune responses (both innate as well as adaptive) to commensal microflora and food antigens are assumed to play pathogenic roles in IBD [2], [3], recent studies have also demonstrated a role for the enterochromaffin (EC) cell in the pathogenesis of this disease.

The EC cell is the most common neuroendocrine cell in the epithelia lining the lumen of the gut and plays a key regulatory role in gut secretion, motility, pain, and nausea [4]. The monoamine neurotransmitter serotonin (5-hydroxytryptamine: 5-HT) has proven central in EC cell regulatory function and these cells synthesize, store, and release the vast majority (95%) of the body’s store of this amine [5]. EC cells function as “taste buds of the gut” and represent sensory transducers responding to mechanical events, luminal acidification, or nutrients such as glucose and short chain fatty acids, bile salt, tastants and olfactants [6]–[13]. In addition, EC cell secretion can be activated by neural, bacterial and immunological input [14], [15]. Specifically, development of IBD is associated with altered EC cell serotonin release [15], [16].

Serotonin is considered to play a role in IBD through activation of immune cell types which express receptors for this amine [15], [17]. *TPH-1* knockout mice respond to chemically-induced colitic agents with a less severe phenotype and delayed onset of disease compared to wild-type mice treated in the same protocol [15]. A variety of other studies [18]–[20] support a role for serotonin in modulating immune signaling and the promotion of interactions between innate and adaptive immune responses within the context of gut inflammation.

Recently, rhythmic mechanical strain that mimics normal bowel movements (mediated by ADORA2B receptors) has been identified to induce EC cell secretion and transcription of EC cell secretory products – responses that are accentuated by neoplasia [21]. We have also demonstrated that gut EC cells are oxygen-responsive and alterations in O_2_ levels differentially activate HIF-1α signaling and serotonin release [22]. This results in alterations in serotonin production and secretion, effects amplified by inflammation. In addition, to the latter, alterations in neuroendocrine signaling as well as activation of hypoxia-mediated responses are features recently identified in a TNBS animal model [23] and in IBD samples through transcriptome analyses [24].

Hypoxia is also strongly associated with an increase in extracellular/mucosal adenosine levels [25] and with stabilization of HIF-1α [26]. HIF-1α induces transcription and increases the activity of 5′ecto-nucleotidase (CD73), the enzyme that converts AMP to adenosine [27]. CD73 also regulates transcription of the ADORA2B receptor while suppressing transcription of the adenosine re-uptake transporters, equilibrative nucleoside transporters 1 and 2 (ENT1 and 2). Furthermore, CD73 decreases the intracellular metabolism of adenosine by suppressing the transcription of adenosine kinase [28]. In IBD, localized hypoxia occurs as a result of chronic inflammation increasing the metabolic needs of the tissue [29], and thus potentially up-regulates the adenosine-ADORA pathway. ADORA2B is the predominant ADORA receptor in colonic mucosa [30] and is also up-regulated by TNFα [31]. Activation of the receptor is thought to regulate cytokine production including IL-10 [32]; colitis is reduced in knockout mice [33], [34] suggesting a protective role.

We hypothesized that the increase in 5-HT observed in IBD may, in part, be due to hypoxia increasing functional HIF-1α which triggers an increase in extracellular adenosine signaling, leading to increased production and secretion of 5-HT via ADORA2B receptor activation. Gut mucosal tissue from IBD patients, isolated EC cells and the well-characterized EC cell line KRJ-1 were studied. This cell line possesses similar properties (e.g. similar signaling pathways, enzyme activity and secretory products) and have similar responsiveness to stimuli, as normal EC cells and is therefore an appropriate model to study 5-HT regulation [13], [21], [35], [36].

## Materials and Methods

### Materials

5′-(N-Ethylcarboxamido) adenosine (NECA) (Sigma-Aldrich, St. Louis, MO), a general adenosine receptor agonist that targets all subtypes (ADORA1, 2A, 2B and 3), curcumin (a HIF-1α inhibitor), SCH442146, a specific A2A receptor antagonist, and MRS1754 hydrate, a specific A2B receptor antagonist were used [37]. The following antibodies for western blot were obtained from Cell Signaling Technology: PKA C-alpha (5842S), MAPK (4695S), pMAPK (4370S), CREB (9197S), pCREB (9198S), Rb IgG (7074S), Mouse IgG (7076S), from Abcam: VMAT-1 (58170) and pTPH-1 (30574), from Alomone labs: A2B adenosine receptor (AAR-003), from Novis Biologicals: TPH-1 (110-57629), from BD Biosciences: HIF-1α (610958), from DAKO: Chromogranin A (CgA: MO869) and from Sigma-Aldrich, β-actin (011M4793).

### Human Samples

Tissue was collected from twenty-one patients (M:F = 12∶9; median age [range] = 53 yr [29–67]). CD tissue (*n* = 12) was obtained from patients who had undergone surgery for CD ileitis (*n* = 3) or colitis (*n* = 9). Only grossly affected tissue was studied. Macroscopically “normal” tissue was obtained from matched samples when available (*n* = 6). All tissue was collected between 2008 and 2013 at Yale University, Department of Surgery following written informed consent from patients *per protocol* (Yale University School of Medicine IRB approval, HIC#0805003870).

### Animals

For the TNBS-colitis model, female Sprague Dawley rats (200–250 g; Taconic) (*n* = 9) were used including controls (vehicle [0.6 ml 50%, ethanol]: *n* = 3), TNBS (29.3 mg/ml, FLUKA; *n* = 3) or TNBS +5-aminosalicylic acid (5-ASA) (4 g/60 ml, volume 1.4 ml; *n* = 3). Volumes were rectally instilled and colitis was confirmed by endoscopy [23]. The study was terminated day 12 (after instillation) with blood and tissue collection. An additional 11 rats were used for EC cell isolation for the antisense studies.

### ADORA2B Knockdown

A 19-mer oligonucleotide antisense corresponding to 461–480 of the rat ADORA2B receptor (NM_01716.1) was designed to induce a steric obstacle for protein translation (Yale Medical School Keck Oligonucleotide Synthesis Facility) [38]. Control nucleotides were prepared with randomized sequence of matching nucleotides per protocol. In these experiments, isolated EC cells [39] were exposed to oligonucleotides (antisensense: TCCCTCTTGCTCGTGTTCC, or control: CTGTTCCGTCCGTTCCCTT –150 pmol) for 12 hrs (FITC-uptake of oligonucleotides was noted as early as 2 hrs within cells, with peri-nuclear uptake complete by 14–16 hrs), and then assessed for mRNA, flow cytometry (receptor expression), secretion and by western blot. These experiments were conducted within 16 hrs following oligonucleotide uptake.

### EC Cell Isolation

EC cells (>98% purity) were isolated from human or rat samples by mucosal stripping, enzymatic digestion, and a combination of Nycodenz gradient fractionation and fluorescence activated cell sorting (FACS) as described [13], [16], [35], [39]. Approximately 1×10^6^ cells were obtained per mucosal sample, a quantity sufficient for real-time PCR, short-term culture and western blots.

### Cell Culture Studies

KRJ-I cells [40] were maintained as floating aggregates in Quantum 263 complete tumor growth medium (PAA) supplemented with penicillin (100 IU/ml) and streptomycin (100 ug/ml). EC cells (normal, IBD or isolated from rat) were maintained in short-term culture (<12 hrs after isolation) under the same conditions. All experiments were performed without antibiotics; the cell line was *mycoplasma* free.

Hypoxic conditions were induced using a modular incubator chamber (MIC-101, Billups-Rothenberg Inc, Del Mar, CA). Briefly, short-term cultured EC cells or cultured KRJ-I cells (48 hrs) were transferred to the humidified hypoxic chamber; the chamber was flushed with CO_2_ for 4 min to maintain hypoxic conditions (0.5% O_2_). KRJ-I cells (4×10^5^ cells/ml, *n* = 6) were seeded in 6 well plates (Falcon, BD, Franklin Lakes, NJ) NECA, curcumin, SCH442146, MRS1754, and DMSO were added to the wells. DMSO was added to the controls to compensate for NECA and MRS1754 being solubilized in DMSO (<0.1% final concentration). Cells were then incubated for 15 minutes. They were then exposed to hypoxia for 0, 15, 30, 60, 120 and 240 mins.

After cells were harvested, whole-cell lysates were prepared by adding 200 µl of ice-cold cell lysis buffer (10× RIPA lysis buffer [Millipore, Billerica, MA], complete protease inhibitor [Roche, Indianapolis, IN], phosphatase inhibitor set 1&2 [Calbiochem, Gibbstown, NJ], 100 mM PMSF [Roche], 200 mM Na_3_VO_4_ [Acros Organics], 12.5 mg/ml SDS [American Bioanalytical, Natick, MA]). Tubes were centrifuged at 12,000 g for 20 min and protein amount in the supernatant was quantified using the BCA protein assay kit (Thermo Fisher Scientific, Rockford, IL).

### Serotonin Secretion

5-HT levels were analyzed using commercially available ELISA assays (5-HT: BA 10-0900; Rocky Mountain Diagnostics) as previously described [13] in supernatant according to the manufacturer’s instructions.

### RLU Studies

The Cignal HIF Pathway Reporter Assay Kit (LUC) (CCS-007L) was used to evaluate HIF signaling in EC cells (human, ADORA2B-antisense treated rat) and in KRJ-I cells. Briefly, the basis of this protocol is transient transfection with a HIF-responsive luciferase construct that encodes the firefly luciferase reporter gene under the control of a minimal (m)CMV promoter and tandem repeats of the hypoxia transcriptional response element (HRE). This is designed to monitor the activity of HIF-regulated signal transduction pathways in cultured cells. Each reporter is premixed with constitutively expressing *Renilla* luciferase, which serves as an internal control for normalizing transfection efficiencies and monitoring cell viability. Short-term cultured CD EC cells (10,000/well) or KRJ-I cells (10,000/well) were transfected per protocol. *Renilla* luciferase activation following ADORA2 activation was measured using the dual luciferase assay (Glomax). The average maximum response per kit is 4 RLU; in these experiments 2 RLU were identified.

### Western Blot Analysis

Analyses were performed on 30 min hypoxia samples to evaluate total TPH, p-TPH, total CREB, p-CREB, total ERK, p-ERK, HIF-1α and PKA. Total protein lysates (20 µg) were denaturated in SDS sample buffer, separated on a Tris-Glycine gel (10%) and transferred to an Immobilon P (PVDF) membrane (Milipore Corporation, Bedford, MA). After blocking (5% BSA for 60 min at room temperature) the membrane was incubated with primary antibodies (Cell Signaling Technology and BD Biosciences) in 5% BSA/PBS/Tween20 overnight at 4°C. The membranes were incubated with the horseradish peroxidase-conjugated secondary antibodies (Cell Signaling Technology) for 60 min at room temperature and immunodetection was performed using the Western Lightning™ Plus-ECL (PerkinElmer, MA). Blots were exposed on X-OMAT-AR films [40], [41]. The optical density of the appropriately sized bands was measured using ImageJ software (NIH, USA).

### RNA Isolation, Reverse Transcription and RT-PCR Analyses

RNA was extracted from mucosa (macroscopically normal human or rat – normal, TNBS and TNBS-treated with 5′ASA), isolated normal and CD EC cells (1×10^6^), isolated normal and ADORA2B-deficient (antisense) rat EC cells, and KRJ-I cells (1×10^6^, *n* = 4–7) using TRIZOL® (Invitrogen, Carlsbad, CA) then cleaned (Qiagen, RNeasy kit, Qiagen, Valencia, CA) and converted to cDNA (High Capacity cDNA Archive Kit, Applied Biosystems, Carlsbad, CA) [36], [42]. RT-PCR analyses were performed using Assays-on Demand™ and the ABI 7900 Sequence Detection System [36], [42]. Primer sets (*HIF-1α* (human and rat) and *ADORA2B* (rat)) were all obtained from Applied Biosystems and PCR mix on gels were performed to confirm presence of single bands for each primer set. PCR Data was normalized using the ΔΔC_T_ method; *ALG9* was used as a housekeeping gene[43] for human, *GAPDH* was used for rat [23].

### Immunostaining

An established immunohistochemical protocol was used to identify target proteins [44], [45]. Briefly, de-paraffinized sections were incubated with a combination of antibodies (mouse HIF-1α 1∶50 and goat polyclonal Chromogranin A 1∶50) overnight at 4°C and then with goat anti-mouse HRP conjugated (1∶25) and donkey anti-goat Alexa Fluor 488 conjugated (1∶25). A Cy-5 tyramide protocol was used to identify HIF-1α. 4′,6-diamidino-2-phenylindole (1∶100) was used for nuclear identification. Bound antibodies were visualized using immunofluorescent microscopy. A total of 10 clinical samples were examined, HIF-1α staining was identified and quantitated as a subset of chromogranin A-positive cells. Crohn’s mucosa was compared to macroscopically normal mucosa.

### Flow Cytometry

Rat EC cells (control or antisense at 12 hrs) were stained with ADORA2B (1∶500) and flow cytometry was conducted on a BD FACS Aria Cell Sorter (BD Biosciences, Bedford MA). Positive cells were identified for unstained, and the two EC cell populations (antisense-treated and control).

### Statistics

Results were expressed as mean±standard error (SEM). All statistical analyses were performed using Prism 4 (GraphPad Software, San Diego, CA). Results were compared between control and stimulated cells using the Mann-Whitney test. A *p*<0.05 was considered significant.

## Results

### HIF-1α Expression in Normal and IBD Mucosa and EC Cells

**Figure 1:**
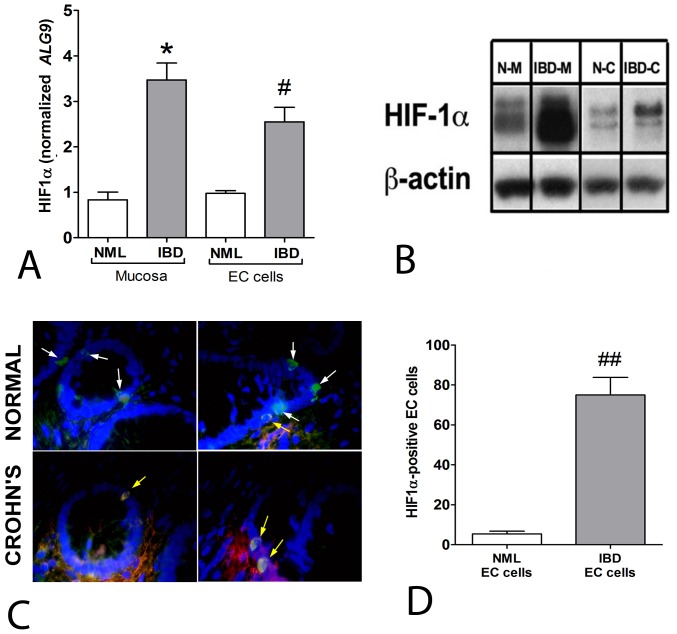
*HIF-1α* transcripts and protein in normal mucosa, IBD mucosa, isolated normal and IBD-EC cells. **1A)** Transcript of HIF-1α was significantly elevated in IBD-associated conditions (mucosa: 3.4±0.63 fold, cells: 2.4±0.41). **1B)** Protein levels were significantly elevated in IBD-associated conditions (mucosa: 12.2±3.4, cells: 2.6±0.36). **1C)** Immunohistochemical staining of HIF-1α and Chromogranin A (white arrows) in normal mucosa and Crohn’s mucosa identified co-staining (yellow arrows) predominantly in IBD mucosa. **1D**) Quantitation identified significantly more enteroendocrine cells to be HIF-1α positive in IBD mucosa. Mean±SEM, *n* = 4–7, *p = 0.03 vs. normal mucosa, ^#^ *p* = 0.04 vs. normal EC cells,^ ##^ *p*<0.001 vs. normal EC cells. DAPI – nuclei (blue), FITC-CgA (green), Cy5-HIF-1α (red), co-localization (yellow). N–M = normal mucosa, IBD-M = IBD mucosa, N–C = normal EC cells, IBD-C = IBD EC cells.

*HIF-1α* transcripts were increased 3.5-fold in IBD mucosa compared to macroscopically normal CD mucosa (**Figure 1A**). A similar pattern was evident at the EC cell level, with CD EC cells demonstrating a ∼2.5-fold increase of *HIF-1α* mRNA compared to normal EC cells. Assessment of protein expression confirmed HIF-1α activation both in mucosa as well as in EC cells isolated from Crohn’s mucosa (**Figure 1B**). Immunofluorescent staining of CgA and HIF-1α (**Figure 1C**) identified double positive cells in the mucosa (yellow arrows), confirming that the HIF-1α-positive cells were enteroendocrine. Significantly more EC cells (∼90%, *p*<0.001) were positive in Crohn’s mucosa than in macroscopically normal mucosa (**Figure 1D**). These results suggest that EC cells are exposed to hypoxia during inflammation and predominantly exhibit an activated hypoxia-mediated signaling pathway (HIF-1α).

### The 5-HT Secretory Pathway

**Figure 2:**
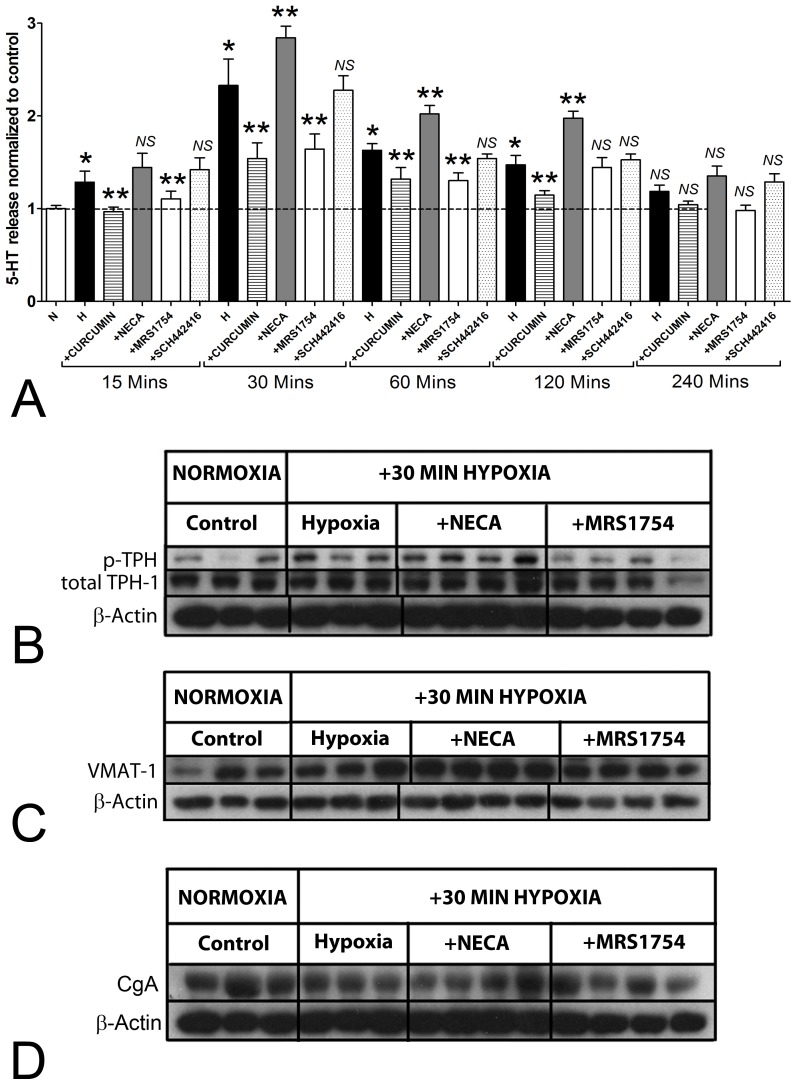
Effect of adenosine on the hypoxia-activated 5-HT pathway in KRJ-I cells. **2A)** 5-HT was increased by hypoxia between 15–120 min, with a maximal effect at 30 min (2.28±0.12 fold). Curcumin and MRS1754 inhibited while NECA augmented secretion at all time points up to 120 mins. SCH442146 had no significant effect. **2B)** Total TPH-1 protein levels were unchanged after 30 min of hypoxia and after NECA or MRS1754 stimulation. Phosphorylated TPH-1 was significantly increased under hypoxia (1.80±0.26), was amplified by NECA (2.66±0.28) and reduced by MRS1754 to baseline. **2C)** VMAT-1 protein levels were significantly increased (2.83±0.31) by NECA during hypoxia and reduced by MRS1754 (2±0.1). **2D)** Chromogranin A protein levels did not change significantly after hypoxia or with the addition of NECA or MRS 1754. Mean±SEM, *n* = 3–8, *p<0.05 vs. control, **p<0.05 vs. hypoxia. NS = not significant.

To evaluate whether adenosine-mediated HIF1α activation increased 5-HT release from EC cells, we examined the effects of hypoxia with and without ADORA2 antagonists on KRJ-I cells over a 4 hr time period. Hypoxia significantly increased 5-HT between 15 and 120 mins with a maximal effect (2.2 fold, *p*<0.05 versus no hypoxia, **Figure 2A**). Curcumin, a known HIF-1α inhibitor through transcriptional repression [46], reversed hypoxia-mediated secretion at all time points. NECA, increased 5-HT release (30–120 min), while MRS1754 but not SCH442146, decreased it (15–120 min). This suggests that 5-HT release by hypoxia is driven, at least in part, by activation of ADORA2B receptors, and that adenosine can modulate 5-HT secretion.

We next evaluated expression of enzymes involved in 5-HT synthesis and vesicle uptake (TPH-1 and VMAT-1) and in secretion per se, chromogranin A (CgA). We focused on 30 mins as this identified the time point at which 5-HT was maximally secreted. Total protein levels of TPH-1 were unchanged by hypoxia at 30 min (**Figure 2B**) but the phosphorylated form of this enzyme, which identifies activated TPH-1, was increased. This was amplified by NECA and inhibited by MRS1754. Analysis of the ratio of activated to total TPH-1 protein identified that this was increased 1.6±0.16 by hypoxia (*p*<0.05 vs. controls) and 2.4±0.2 (by NECA: *p*<0.05 vs. hypoxia) and was reduced to 0.91±0.17 (by MRS, *p*<0.05 vs. hypoxia). VMAT-1 functions to accumulate cytosolic monoamines, like 5-HT, into secretory vesicles [47]. No significant changes were noted for VMAT-1 by hypoxia, but levels were significantly elevated by NECA (2.83±0.07, *p*<0.05) (**Figure 2C**), indicating that adenosine signaling activates 5-HT uptake into vesicles. CgA is important for granulogenesis and secretion in neuroendocrine cells [48]. No alterations were identified (**Figure 2D**). We interpret this to indicate that hypoxia and adenosine directly activate pathways associated with the formation of components essential to neuroendocrine secretion.

### Directly Linking ADORA2B and HIF-1α

**Figure 3:**
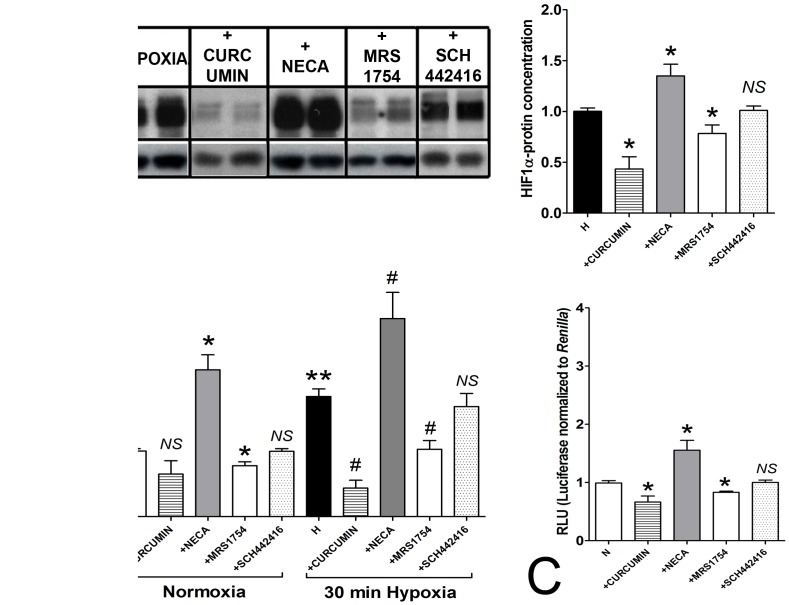
HIF-1α, adenosine signaling and HRE activation. **3A)** Curcumin inhibited (022±0.04 fold) while NECA (1.24±0.06 fold) stimulated and MRS1754 (0.74±0.02) inhibited HIF-1α protein levels compared to 30 minutes hypoxia in KRJ-I cells. SCH442146 had no effect. **3B)** Transient transfection with *Renilla* luciferase-encoding constructs in KRJ-I cells. Under normoxic conditions, NECA amplified activation of luciferase (RLU) while MRS1754 inhibited this. Curcumin and SCH442416 had no effect. Hypoxic conditions activated RLU (1.81±0.12), which was inhibited by curcumin and MRS1754 and amplified by NECA. **3C)** In IBD-EC cells, RLU was elevated in normoxic conditions. This could be reduced by curcumin and MRS1754 and amplified by NECA. Mean±SEM, *n* = 3–7, *p<0.05 vs. 30 min hypoxia or control, **p<0.05 vs. normoxic cells, ^#^ *p*<0.05 vs. 30 min hypoxia. NS = not significant.

We next evaluated whether adenosine could activate HIF-1α. In KRJ-I cells, 30 min hypoxia-induced HIF-1α protein expression was reversed by curcumin (**Figure 3A**). Pre-treatment of cells with NECA (15 mins prior to hypoxic challenge) significantly increased HIF-1α protein (∼1.3-fold) while MRS1754 significantly decreased this (∼0.8-fold). SCH442146 had no effect.

To evaluate HIF-1α mediated signaling we undertook transient transfection with a HIF-responsive firefly luciferase construct under HRE-transcription control (*Renilla* luciferase-constructs) in KRJ-I cells (**Figure 3B**) and in IBD-EC cells (**Figure 3C**). In KRJ-I cells NECA activated luciferase (RLU) under normoxic conditions while MRS1754 inhibited this; no effect was noted for SCH442146. Under hypoxic conditions, NECA amplified luciferase activity (3-fold, *p*<0.05 vs. hypoxia) which was inhibited by MRS1754 but not by SCH442146. In IBD-EC cells (which have an activated HIF-1α–**Figure 1A–C**), activation of HRE-mediated transcription was amplified by NECA (∼1.5-fold RLU) under normoxic conditions and inhibited by MRS1754. These results suggest that adenosine, similar to a reduction in O_2_, can increase HIF-1α protein levels and induce HRE-signaling which is mediated via the ADORA type 2B receptor.

### Analysis of Secretion-associated Signaling Pathways

**Figure 4:**
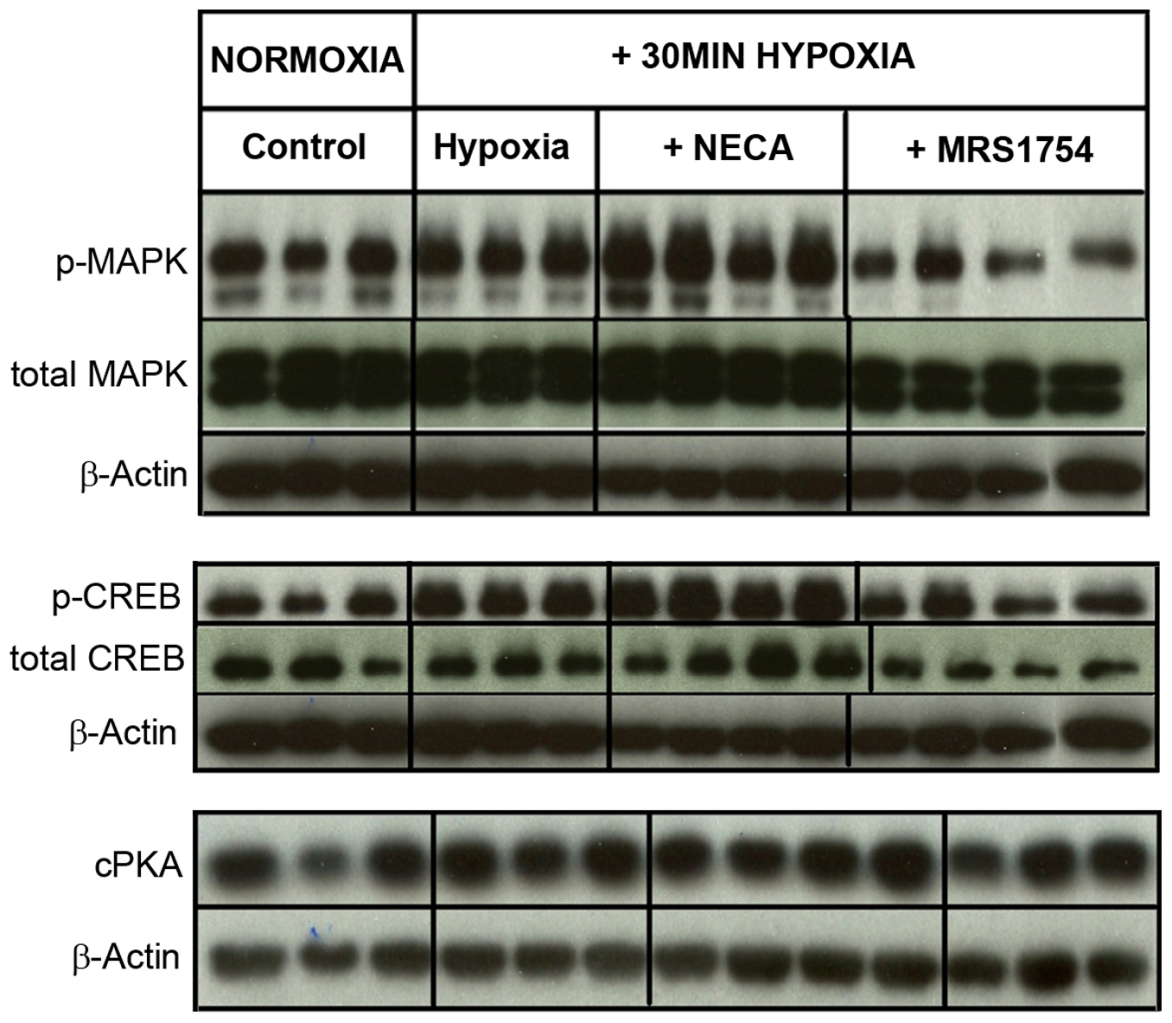
Effects of hypoxia and adenosine on signaling pathways. **4A)** Phosphorylated MAPK was not significantly altered by hypoxia but was amplified by NECA (1.29±0.06)and reduced by MRS1754 (0.40±0.09); total MAPK protein amount was unchanged. **4B)** Neither total CREB nor phosphorylated CREB were altered after 30 min of hypoxia. NECA, however, stimulated p CREB compared (1.55±0.07); this was reduced by MRS1754 (0.7±0.12). **4C)** Free catalytically active PKA protein levels were unchanged after 30 min of hypoxia, as well as after NECA and MRS1754. Mean±SEM, *n* = 5–8.

We next evaluated whether signaling pathways related to 5-HT release [21], [49] were altered by hypoxia and adenosine. MAPK kinease phosphorylation, although unchanged by hypoxia, was amplified by NECA, and reduced by MRS-1754 (**Figure 4A**). This suggests that the ADORA2B receptor under hypoxic conditions regulates MAP kinase activity – a known regulator of 5-HT secretion and TPH-1 phosphorylation [13], [35].

CREB is an important transcription factor for *TPH-1* transcription [50]–[52]. While not significantly increased by hypoxia, the phosphorylated form was significantly increased by NECA (**Figure 4B**), an effect that was reversed by MRS1754. This suggests a role for ADORA-mediated signaling in the regulation of hypoxia-mediated *TPH1* transcription.

PKA, which mediates the adenosine signal for 5-HT production and secretion under mechanical stress [21], was not significantly changed by hypoxia and was not altered by either NECA or MRS1754 (**Figure 4C**) suggesting that this pathway does not regulate 5-HT secretion under these conditions.

### Mechanistic Analysis of Hypoxia-adenosine Signaling Pathways in EC Cells

**Figure 5:**
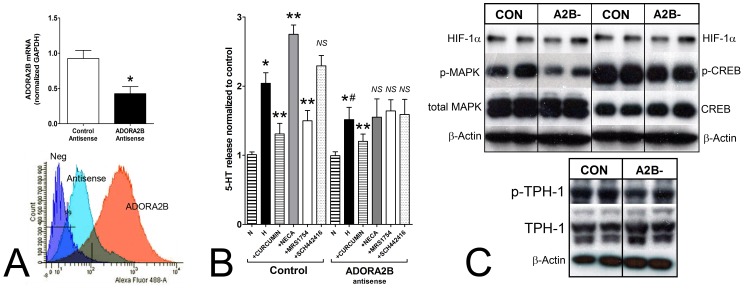
Analysis of 5-HT release and signaling pathways in ADORA2B-deficient rat EC cells. **5A)** A 12 hr antisense approach inhibited ADORA2B transcript (real-time PCR) and protein expression (membrane-bound, flow cytometry) in isolated rat EC cells. Membrane expression was reduced to an estimated 5% of non-targeted cells. **5B)** In non-targeted cells, 5-HT release was elevated by hypoxia and inhibited by curcumin. Adenosine signaling responses were similar to human EC cells. Antisense reduced hypoxic-mediated responses (compared to controls) and ameliorated cell responses to NECA and MRS1754. **5C)** Western blot identified decreased pMAPK (0.56±0.09), pCREB (0.75±0.11) and pTPH-1 (0.63±0.07) in antisense treated cells confirming down-regulation of these pathways with loss of ADORA2B expression under hypoxic conditions. Mean±SEM, *n* = 3. *p<0.05 vs. control, **p<0.05 vs hypoxia, ^#^ *p*<0.05 vs. hypoxia (in non-antisense treated cells). NS = not significant.

We used an antisense approach to mechanistically dissect the pathways associated with hypoxia-adenosine signaling in isolated rat EC cells. ADORA2B antisense decreased transcription (∼50%) and was associated with a significant and almost complete reduction in ADORA2B membrane protein expression (**Figure 5A**). These cells could respond to hypoxia with 5-HT release, but this was significantly lower than cells with normal ADORA2B expression (**Figure 5B**). The latter cells responded similar to human EC cells with adenosine-regulated hypoxia-mediated 5-HT release, while the antisense-treated cells had largely lost adenosine mediated effects. At a protein level, we identified reduced pMAPK, pCREB and pTPH-1 in antisense treated hypoxic cells (**Figure 5C**) identifying that these are the principle pathways involved in adenosine-mediated 5-HT secretion.

### Hypoxia and ADORA2B in an Animal TNBS-induced Colitis Model

**Figure 6:**
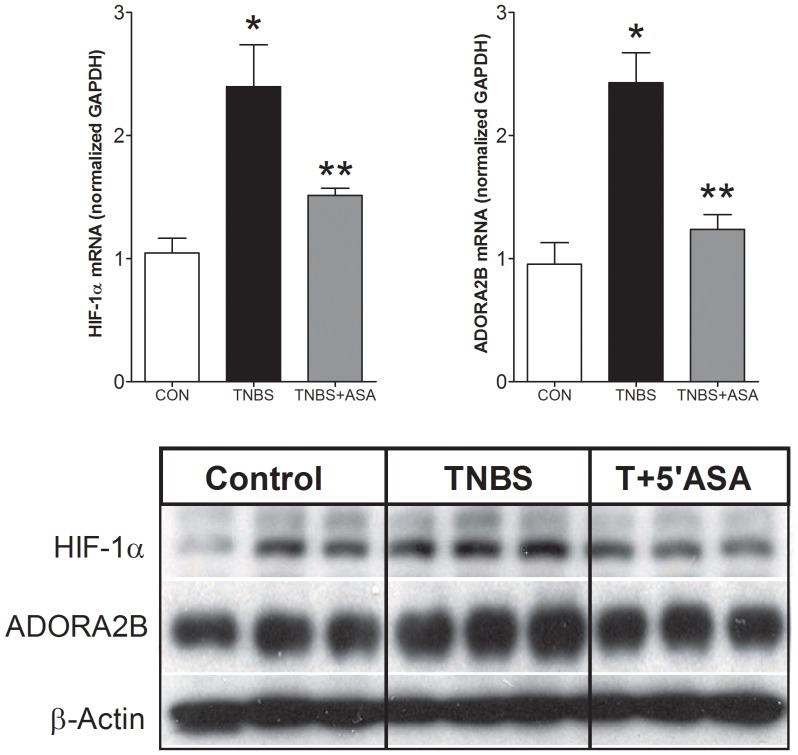
Analysis of HIF-1α and ADORA2B in mucosa from the animal TNBS-induced colitis model with or without 5-ASA-treatment. 6A) TNBS was associated with a significant elevation in HIF-1α and ADORA2B transcripts. Treatment with 5-ASA reversed these values to those similar to untreated rats. 6B) Protein expression was elevated by TNBS (HIF-1α: 1.68±0.11; ADORA2B: 1.37±0.09) and reversed by 5-ASA in mucosa. Mean±SEM, *n* = 3. *p<0.05 versus control, **p<0.05 versus TNBS.

Finally, we evaluated expression of HIF-1α and ADORA2B in intestinal mucosa from a rat animal TNBS-induced colitis model and evaluated the effect of 5-ASA. A real-time PCR analysis confirmed significant upregulation of HIF-1α and ADORA2B mRNA by TNBS (**Figure 6A**). The expression levels were decreased by 5-ASA treatment. Protein levels followed a similar expression pattern (**Figure 6B**). We interpret these data to confirm activation of a hypoxia-adenosine pathway in a colitis model – similar to observations in clinical samples.

## Discussion

IBD is associated with abnormalities in the pro-inflammatory 5-HT system. Previously, we have demonstrated that LPS and IL1β induce a significantly elevated 5-HT response in IBD mucosa [16]. This may partly explain the hypersecretion of 5-HT noted in the IBD disease process. As IBD mucosa is also exposed to significant hypoxic stress, we examined the effects of hypoxia on EC cell 5-HT secretion. We have identified that hypoxia induces 5-HT secretion from EC cells and that this can be reversed by targeting HIF-1α. HIF-1α is also associated with increased mucosal adenosine availability, a known regulator of 5-HT production and secretion [22]. Based on these observations, we investigated the relationship between hypoxia, the ADORA signaling system and 5-HT production/secretion in EC cells from CD and normal mucosa to evaluate whether these factors are involved in the amplified 5-HT production noted in IBD.

We demonstrated that HIF-1α was increased both at the mRNA and protein levels in IBD mucosa, in an animal model of colitis and in IBD-EC cells. The latter almost predominantly (∼90%) expressed a hypoxia activated phenotype and active HRE signaling. These data confirm that the inflamed mucosa is under hypoxic stress [53], [54], and that EC cells, in particular, are hypoxia-activated. We have previously identified increased ADORA2B receptors both at protein and transcript levels in EC cells isolated from IBD patients compared to controls [21]. In the current study, we identified that adenosine amplified HIF-1α expression and activity, which suggests that it can act as a positive feedback mechanism for 5-HT synthesis and release.

Activating ADORA signaling via NECA increased 5-HT release which could specifically be inhibited by MRS1754 identifying that, under low oxygen mucosal levels such as found with hypoxia, EC cell secretion is regulated by adenosine and specifically, via activation of ADORA2B receptors. The inhibitory effect of MRS1754 was complete for the time period 15–90 mins but at 120 mins, we could identify no inhibition by this agent. This suggests that the adenosine (ADORA2B):hypoxic signaling pathway occurs early (within 30 mins) and that other ADORA receptors may play a role later in hypoxia-mediated 5-HT release; it is unlikely that this is ADORA2A. A combination of HIF-1α signal activation and adenosine-mediated ADORA2 signaling appears to play important roles in EC cell 5-HT secretion in IBD.

HIF-1α itself may directly regulate transcription of the rate-limiting enzyme in 5-HT synthesis TPH-1. The promoter region of this gene encodes hypoxia responsive elements (HRE) [55]; hypoxia may therefore directly increase 5-HT production by increasing *TPH-1* expression. Transcriptional alterations in TPH-1 were not consistently identified in our current study (*data not shown*) suggesting the major route regulating 5-HT release occurred at the level of protein regulation and via pathways cross-activated by HIF-1α signaling.

We have previously demonstrated that the adenosine/ADORA2B/cAMP/PKA/CREB pathway plays a pivotal role in regulating 5-HT secretion from the EC cell when subject to mechanical stress [21]. In the current study, we demonstrated that the activation of the ADORA2B receptor was important for the increased levels of 5-HT noted during hypoxia. The intracellular signaling pathways involved in the hypoxic response, however, exhibit differences to that identified for EC cell mechano-responsivity. We failed to detect differences in PKA suggesting that activation of cAMP-responsive pathways is not PKA-regulated under these conditions. In contrast, we identified ADORA2B mediated activation of MAPK signaling as well as increased phosphorylation (and thereby activation) of TPH-1. TPH-1 is a MAPK target and it is likely, under hypoxic conditions, that MAPK phosphorylates and thereby activates this enzyme, leading to an increase in 5-HT synthesis. Antisense approaches mechanistically confirmed roles for both MAPK as well as TPH-1 in EC cells under hypoxic conditions.

Other factors involved in 5-HT secretion were altered by adenosine-ADORA2B signaling. Specifically, protein levels of the rate-limiting enzyme involved in 5-HT vesicular accumulation, VMAT1, were increased. We postulate that the excess 5-HT produced by activated TPH-1, is actively transported into vesicles which then provides a large pool for release. We interpret this to demonstrate that vesicle maturation [47], [48], is directly regulated by adenosine under hypoxic conditions.

Adenosine plays a complex role in IBD. Apart from activating EC cell 5-HT secretion, this nucleotide can also decrease SERT activity [56]; the combination resulting in a increased mucosal 5-HT signal. One prediction, given the pro-inflammatory activity of 5-HT, is an exacerbation of colitis under these conditions. Other studies have demonstrated an increased severity of colitis in ADORA2B−/− mice suggesting that targeting this receptor may be a potential therapeutic target. Currently, loss of ADORA2B is considered to result in down-regulation of IL10 production, with accentuation of colitis. Our studies indicate that loss of ADORA2B decreases 5-HT and, presumably, the mucosal aminergic signal.

Elevated mucosal adenosine, induced in hypoxic conditions or during abnormalities in peristalsis, may activate EC cell 5-HT synthesis and release, and reduce enterocyte-mediated SERT production with a resultant overall increase in 5-HT pro-inflammatory signaling. In the context of ADORA signaling, our studies and others, highlight both the complexity of the mucosal pathways involved in IBD as well as the importance of delineating individual cell signaling. This may help better direct targeted therapy.
