# Correction: The Stimulatory Adenosine Receptor ADORA2B Regulates Serotonin (5-HT) Synthesis and Release in Oxygen-Depleted EC Cells in Inflammatory Bowel Disease

**DOI:** 10.1371/annotation/99ad70ea-d3ca-485c-a1b4-50c107941c94

**Published:** 2013-07-30

**Authors:** Rikard Dammen, Martin Haugen, Bernhard Svejda, Daniele Alaimo, Oystein Brenna, Roswitha Pfragner, Bjorn I. Gustafsson, Mark Kidd

The name of the first author was spelled incorrectly. The correct name is: Rikard Dammen. 

The correct citation is: Dammen R, Haugen M, Svejda B, Alaimo D, Brenna O, et al. (2013) The Stimulatory Adenosine Receptor ADORA2B Regulates Serotonin (5-HT) Synthesis and Release in Oxygen-Depleted EC Cells in Inflammatory Bowel Disease. PLoS ONE 8(4): e62607. doi:10.1371/journal.pone.0062607. 

